# Robotic surgical systems in maxillofacial surgery: a review

**DOI:** 10.1038/ijos.2017.24

**Published:** 2017-06-29

**Authors:** Hang-Hang Liu, Long-Jiang Li, Bin Shi, Chun-Wei Xu, En Luo

**Affiliations:** 1 State Key Laboratory of Oral Diseases, National Clinical Research Center for Oral Diseases, West China Hospital of Stomatology, Sichuan University, Chengdu, China

**Keywords:** head and neck, maxillofacial surgery, oral surgical procedures, robotic surgery

## Abstract

Throughout the twenty-first century, robotic surgery has been used in multiple oral surgical procedures for the treatment of head and neck tumors and non-malignant diseases. With the assistance of robotic surgical systems, maxillofacial surgery is performed with less blood loss, fewer complications, shorter hospitalization and better cosmetic results than standard open surgery. However, the application of robotic surgery techniques to the treatment of head and neck diseases remains in an experimental stage, and the long-lasting effects on surgical morbidity, oncologic control and quality of life are yet to be established. More well-designed studies are needed before this approach can be recommended as a standard treatment paradigm. Nonetheless, robotic surgical systems will inevitably be extended to maxillofacial surgery. This article reviews the current clinical applications of robotic surgery in the head and neck region and highlights the benefits and limitations of current robotic surgical systems.

## Introduction

Maxillofacial surgeries have conventionally been performed with large incisions, either *via* a transmandibular or a transpharyngeal approach, because of the complicated anatomy and limited surgical space. These procedures typically result in significant surgical morbidity, speech dysfunction and dyspepsia from the dissection of large amounts of normal tissue. However, minimally invasive surgical technologies have evolved dramatically over the past two decades since Mouret^1^ completed the first laparoscopic cholecystectomy in 1987. This technique allows surgeons to access tissue through a few small incisions instead of a large incision. The focus of these procedures is now on preserving function, reducing postoperative morbidity and improving quality of life.

Nevertheless, the use of minimally invasive surgery (MIS) in maxillofacial surgery has posed challenges related to neurovascular control, illumination of the surgical field and protection of the surrounding structures. In 2000, Steinier^2^ advocated transoral laser microsurgery, which demonstrated superior results. Unfortunately, this approach obstructs the line of sight, as visualization is provided by merely a microscope. With this approach, sufficient exposure of the surgical field cannot be obtained, and resection is not possible in the cranial and axial axes. To overcome these limitations, robotic surgical systems were innovated and introduced into surgical practice. Transoral robotic surgery (TORS) was proposed and first applied clinically in maxillofacial surgery by McLeod and Melder^3^ to excise a vallecular cyst. This procedure was approved by the US Food and Drug Administration (FDA) in 2009 for use in stage T1 and T2 oropharyngeal cancer. Since that time, robot-assisted maxillofacial surgery has been growing steadily in popularity. Taking inspiration from its use in other surgical fields, the benefits to surgeons include a three-dimensional magnified view, precise movements, bimanual operation with articulated arms and suppression of tremor, which enhances the surgeon's physical capabilities. Thus, procedures with robotic assistance can be performed with less blood loss, fewer complications, shorter hospital stays and better cosmetic results than standard open techniques.^4^

Hence, robotic surgery may hold promise in the treatment of craniofacial conditions, such as head and neck neoplasms, cleft palate and craniofacial asymmetry, among others. In this review, we summarize the current applications of robot-assisted maxillofacial surgery.

## History of robotic surgical systems

**Figure 1:**
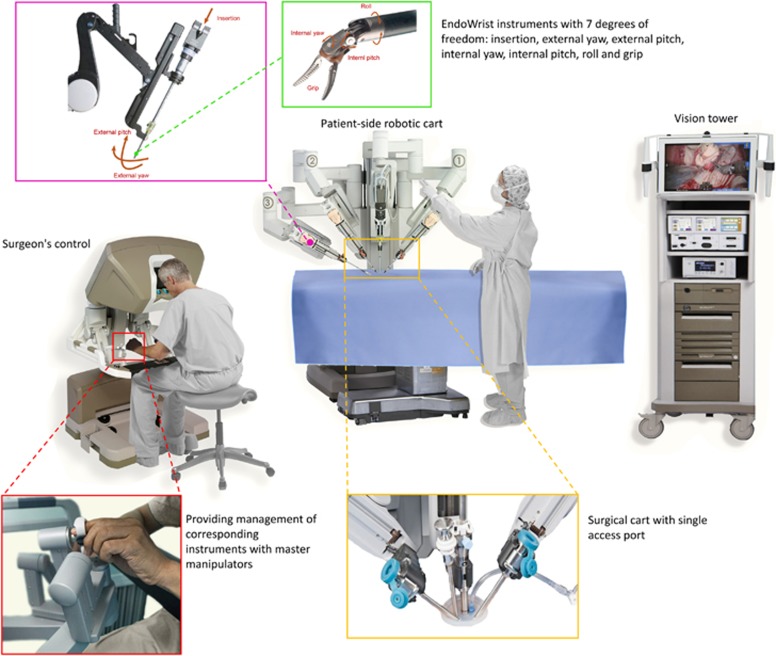
**Robotic surgery operating room schematic.**

For decades, robots and surgery have been developing along two independent paths. During the late 1980s and early 1990s, endoscopic techniques were booming, and limitations were being reached as well. Subsequently, the potential capability of telerobotics in MIS was well recognized. However, robots and surgery only reached a safe enough stage for their combination *via* telemanipulation for surgical innovation in the last few years. The robotic surgical system is truly an information system rather than a machine, and it can be simply divided into input, analysis and output. A human is interposed between the input and output instead of a computer in case there are any unexpected events or anatomy during surgery, and these components serve as a teleoperation system.^5^ The input side consists of several chemical and biologic sensors and imagers, and there are various devices on the output side, such as manipulators and lasers, to contact organs and tissues. The robotic surgical system was manufactured to overcome the limitations of laparoscopic surgery, including tremor, fatigue, 2D imaging and a limited range of freedom. Additionally, robotic surgery can also be described as an ability to enable surgical interventions *via* the application of telecommunications and robotic systems, where the patient and surgeon are separated. Since Puma 560,^6^ the first robotic surgical system was introduced in the mid-1980s to orient a needle for brain biopsy, three generations of systems have followed. Generation I: CMI’s Automated Endoscopic System for Optimal Positioning (AESOP). AESOP, a voice-controlled robot, was developed to serve as a stable camera platform and not multi-arm units. AESOP eliminates the need for an extra surgical assistant, and AESOP 1000 was approved by the FDA for use in surgery in 1995. Even though AESOP was widely applied in various surgical settings, including cardiology, urology and gynecology, until 1999,^7^ there were several deficiencies. In addition, the robotic system required a few alterations to cooperate with surgeon’s style of operation. Generation II: Telerobot Zeus. Zeus was a kind of master-slave teleoperator between the surgeon and the patient-side manipulator. Zeus was introduced in 1995 to provide improved precision for the laparoscopic surgeon, and it was approved by the FDA in 2000. Zeus consists of an AESOP robotic scope and two additional manipulators to hold the operating instruments, and the three arms are mounted to an operating table. It had the advantages of remote control, three-dimensional visualization and tremor suppression. In addition, this telemanipulator allowed a surgeon to perform surgical procedures from a remote region, such as hospital-to-hospital settings. However, it was no longer technically supported once the da Vinci surgical system began being used worldwide. Generation III: da Vinci surgical system. Comparatively, the da Vinci system aimed at recreating the feeling of open surgery and was preferred by the open surgeon, while the Zeus system was primarily adopted by the laparoscopic surgeon. The initial da Vinci robot was invented in 1999 by Intuitive Surgical, and it consists of three major parts: a surgeon’s console, a robotic cart on the patient’s side and a high-definition 3-dimensional vision tower.^8^ The surgeon’s console enables management of the corresponding instruments with master controls, and it was derived from part of the M7 system developed by Stanford Research Institute (SRI)—a surgical robot for open surgery.^5^ The surgeon can operate from a comfortably seated position while having a high-definition real-time view inside the patient. The patient-side surgical cart consists of three or four arms that were originally developed from the Black Falcon system: one arm handles the endoscopic camera (passes through a 12-mm trocar), while the other two or three arms hold the EndoWrist instruments (pass through 8-mm trocars), which provide enhanced degrees of freedom and excellent 3D imaging. This permits large-scale movement in surgery, such as the movements needed for dissecting and suturing. Moreover, the camera used in the system provides a true-to-life stereoscopic image of the patient’s anatomy, which is transmitted to both the surgeon’s console and the vision tower beside the surgical assistant.^8^ The vision tower provides a broad perspective and visualization of the procedure to the surgical assistant at the patient’s side (Figure 1). Recently, several developments have been made. First, the da Vinci Si system was manufactured to support two consoles operating in concert with one patient-side robot; thus, an instrument “give-and-take” was made available. Second, 5-mm-diameter instruments are now available. Third, in the da Vinci Xi robot, the laser targeting system can simply point the scope at the target anatomy, and a smaller robotic arm and footprint along with improved articulation provide increased flexibility and decreased arm collisions. Fourth, a single port robotic technique, which is less invasive than procedures with several access ports, has already been launched and is on the market, but it has unfortunately not been applied in maxillofacial surgery. Apart from those mentioned above, there are several other robotic surgical systems, including ROBODOC, Computer-Assisted Surgical Planning and Robotics (CASPAR), Robotic Arm Interactive Orthopedic System (MAKO Surgical Corp RIO) and so forth, that have been generally applied in orthopedic surgery, such as arthroplasty.^5^

Overall, the da Vinci surgical system is currently considered the most successful robotic surgery system; it has been widely utilized in multiple anatomic regions since Pasticier *et al.*^9^ first utilized it in radical prostatectomy. This system was first used in maxillofacial surgery in 2005, and it was approved by the FDA in 2009. Currently, the da Vinci robot is used for almost all surgical procedures performed in the head and neck region.^3^

## Clinical applications of robotic surgery in the head and neck

### Search methods

**Figure 2:**
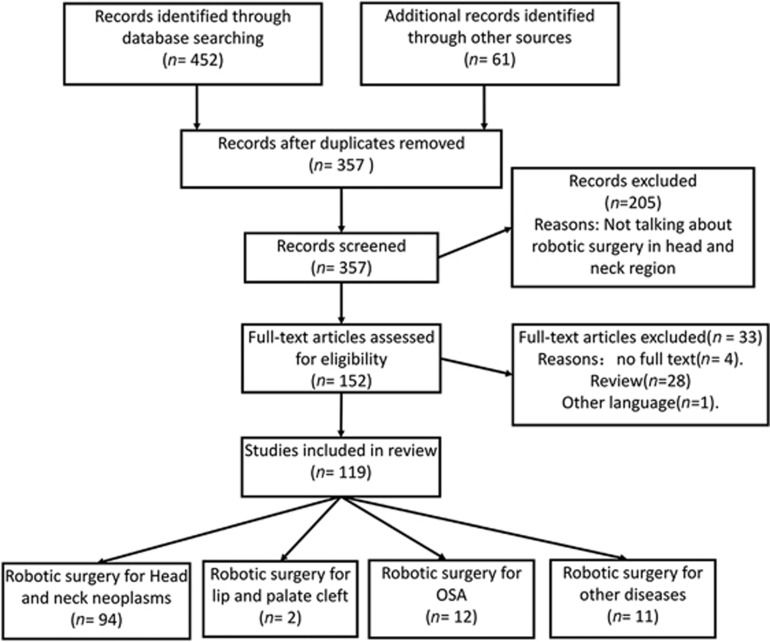
**Diagram of article retrieval.**

The literature search was performed using the Cochrane Central Register of Controlled Trials (CENTRAL; 2016), MEDLINE (*via* PubMed, 1948 to September 2016), Embase (1974 to September 2016), the China National Knowledge Infrastructure (CNKI; 1979 to September 2016) and China Biology Medicine (CBM; 1978 to September 2016). Gray databases, such as OpenGrey and Sciencepaper Online, were also searched. Manual searches were also conducted in relevant Chinese journals, and reference lists of relevant articles were reviewed. To find ongoing clinical trials, the World Health Organization International Clinical Trials Registry Platform was searched. MeSH heading words and free text words were combined. They included “Robotics,” “Operation, Remote,” “Oral Surgical Procedures,” “Oral Surgery” and “Head and Neck Neoplasms.” Language was restricted to Chinese and English. As a result, a total of 503 studies were identified; of these, 119 that were associated with the application of robotic surgery in the head and neck region were included in this review (Figure 2).

### Clinical applications

The development of a robotic surgical system for maxillofacial surgery has been relatively delayed because of the limited surgical field and compact surrounding anatomy. The first application of a robotic surgical system in maxillofacial tumors was reported by Haus *et al.*^10^ for resection of the submandibular gland in animal models. Since that time, the use of robotic surgery for head and neck diseases has been gradually increasing. Currently, the chief indications for robotic surgery in the head and neck region are (1) removal of head and neck neoplasms or cysts that can be sufficiently exposed *via* a robotic approach; (2) therapeutic and selective neck dissection; and (3) obstructive sleep apnea syndrome (OSAS). Meanwhile, tumors with jaw or internal carotid artery invasion are not currently suitable for robot-assisted resection.^10^

#### Head and neck neoplasms

Head and neck neoplasm is a group of neoplasms that arise from the oral cavity, pharynx, larynx, sinuses or salivary glands, among others. Head and neck cancers are regarded as the sixth most common malignancy and ninth most frequent cause of death worldwide; ~529 500 new patients are diagnosed annually, and head and neck cancers are responsible for 3.6% of cancer-specific deaths.^11^ In high-risk countries (that is, India, Sri Lanka, Bangladesh and Pakistan), oral cavity cancer has the highest incidence of the head and neck cancers and is increasing in incidence.^12^ The average 5-year survival rate of head and neck cancer following diagnosis in the developed world is 42–64%, and the 1-year survival rate of advanced oral cavity cancer is <50%.^13^ Currently, surgery is frequently applied as a treatment in most head and neck cancers. However, surgery can be particularly difficult if the tumor is near the larynx, which might result in dysphasia. Of these surgeries, robotic surgery allows the surgeon to remove tumors with minimal damage to normal tissues, and it gives patients as much speech and swallowing function as possible postoperatively. Specific clinical applications of robotic surgery in head and neck neoplasms are presented below.

**Oral cavity, oropharynx, nasopharynx and laryngopharynx**

On the basis of preclinical experiments, robot-assisted surgery for the excision of a vallecular cyst was first performed by McLeod and Melder^3^ in 2005, with no complications experienced. Later, O’Malley and colleagues^14^ reported the technical feasibility of robot-assisted surgery for base of tongue (BOT) neoplasm resection; Weinstein and colleagues^15^ successfully performed a robot-assisted radical tonsillectomy in 2007 after cadaveric robotic surgery. With this much groundwork completed, several studies subsequently focused on the application of TORS in various types of neoplasms, including squamous cell carcinoma,^16, 17, 18, 19, 20, 21, 22, 23, 24, 25, 26, 27, 28, 29, 30, 31, 32, 33, 34, 35, 36, 37, 38, 39, 40, 41, 42, 43, 44, 45, 46, 47, 48, 49, 50, 51, 52, 53, 54, 55, 56, 57, 58, 59^ mucoepidermoid carcinoma,^16, 35, 43, 50, 60, 61^ malignant melanoma,^62^ synoviosarcoma,^33, 63^ adenoid cystic carcinoma,^33, 35, 43, 50, 60, 64^ pleomorphic adenoma,^32, 35, 47, 65^ lipoma^33^ and neurilemmoma.^64^

Several studies have demonstrated that robotic surgery for primary or recurrent neoplasms in the oral cavity, oropharynx, nasopharynx and laryngopharynx has superior functional recovery; higher rates of negative margin, recurrence-free survival, disease-free survival and overall survival; and a lower risk of hemorrhage, gastrostomy tube and tracheostomy tube dependence, and other intraoperative or postoperative complications than conventional open surgery or radiochemical therapy.^38, 52, 66, 67, 68^ However, it is also worth noting that Blanco *et al.*^47^ reported an application of TORS in the treatment of recurrent oropharynx squamous cell carcinoma, in which three of four patients experienced postoperative regional or distal transference. Furthermore, TORS appeared to be more effective in the detection and diagnosis of unknown primary tumors than conventional methods, including computed tomography, positron-emission tomography and directed biopsies, especially for human papillomavirus (HPV)-positive patients.^51, 55, 56, 57, 58, 59^

In addition to the factors mentioned above, other aspects of robotic surgery were assessed. For instance, HPV is one of the most important known risk factors for oropharynx cancer. It is widely accepted that HPV-positive patients with head and neck cancers may have a better prognosis than patients who are HPV-negative. Cohen *et al.*^69^ found that TORS may provide similar surgical and oncologic outcomes to HPV-negative patients, such as negative resection margin; local, regional and distant disease recurrence rates; and disease-free and overall survival rates that are comparable to those of HPV-positive patients; however, other surgeons^24, 42, 43^ held different opinions. Blanco *et al.*^47^ and Olsen *et al.*^28^ determined that the 2-year disease-free survival rate of HPV-positive patients was higher than that of HPV-negative patients, and Quon *et al.*^46^ study showed that HPV-positive patients have a higher positive margin rate. Regarding postoperative quality of life, swallowing and speech functions decreased significantly 3–6 months after TORS and recovered to the preoperative state 1 year later.^23, 70^ Furthermore, the study by Park *et al.*^38^ showed that robotic surgery resulted in significantly decreased postoperative pain and anxiety and a better appetite compared to open surgery. Moreover, the time to functional recovery seemed to be associated with preoperative T stage, tumor location, tumor size, status of tumor (primary or recurrent) and pretreatment M.D. Anderson Dysphagia Inventory (MDADI) score.^19^ Robotic surgery allows surgical instruments to be mounted on the robotic arms; some studies showed that dissection with a laser may provide better surgical outcomes in terms of hemorrhage, intraoperative pharyngotomy, postoperative pain and operation time compared to electrocautery.^34, 49^ Abel *et al.*^34^ proposed that this difference might be related to decreased collateral thermal damage using the laser.

**Parapharyngeal space.**

The parapharyngeal space is a potentially deep and anatomically compact space in the head and neck that contains important structures, including the internal carotid artery and cranial nerves IX, X and XI. Traditionally, the extended facial recess approach, transcochlear approach and transtemporal–infratemporal fossa approach were associated with tumors in this area.^71^ However, these approaches seemed to be associated with significant degrees of morbidity as well as visible scars. O’Malley and Weinstein^72^ first performed robot-assisted resection of a benign neoplasm in the parapharyngeal space based on cadaveric and animal robotic surgery. Several subsequent reports showed favorable results, such as short hospital stays, quick functional recovery and a lack of significant complications, when parapharyngeal neoplasms (squamous cell carcinoma, lipoma, pleomorphic adenoma, adenoid cystic carcinoma, cartilaginous tumor and neurilemmoma) were removed using the robot.^36, 61, 73, 74, 75^ Chan *et al.*^76^ reported that 24% of patients with pleomorphic adenoma experienced unexpected capsule breakage or neoplasm fracture during surgery, potentially resulting from an inability to safely grasp the tumor, sharp instruments and a lack of tactile and haptic feedback.

**Thyroid gland and mediastinal parathyroid.**

Bodner *et al.*^77^ described the first use of a robotic surgical system for mediastinal parathyroid resection *via* a transaxillary incision in 2004 and showed that transaxillary robotic surgery is a minimally invasive, effective and safe procedure. Later, Lewis *et al.*^78^ and Miyano *et al.*^79^ demonstrated the feasibility of transaxillary robotic thyroidectomy. No significant bleeding or edema occurred intraoperatively or postoperatively. Recently, Byeon *et al.*^80^ performed robotic retroauricular thyroidectomy for clinically suspicious papillary thyroid carcinoma. Other previous studies found that robotic thyroidectomy *via* a retroauricular incision is a safe, technically feasible approach with satisfactory cosmetic results.^81, 82, 83, 84, 85, 86^ However, their results indicated that this approach required a longer operative time, longer hospitalization and longer postoperative drainage than endoscopic surgery and open surgery because of the remote access.

In addition, a lingual thyroglossal duct cyst was also excised using a robotic surgery system *via* a transoral approach or a retroauricular approach without complications or recurrence.^87, 88, 89^ A lingual thyroglossal duct cyst is a congenital fibrous cyst that forms from a persistent thyroglossal duct, which was conventionally dissected *via* a transcervical approach. However, the traditional surgery was always associated with an undesirable scar in the neck and a high relapse rate. In Kim *et al.*^89^ opinion, the 3-dimensional, magnified visualization of the robot resulted in less damage to the surrounding normal tissues, reduced intraoperative bleeding and infection, and the ability to ligate the tract after carefully tracing it.

**Salivary glands.**

Submandibular gland tumors were traditionally excised *via* a transcervical approach, which always left a visible scar, and possibly even hypertrophic scarring in the neck. In comparison, on the basis of its guaranteed curative effect, robotic resection of the submandibular gland through a retroauricular approach or modified face-lift approach can produce an invisible scar, making it more acceptable to patients.^90, 91, 92, 93^ The study by Yang *et al.*^93^ showed that gland-preserving robotic surgery has a potentially lower risk of intraoperative hemorrhage, positive margins and postoperative functional nerve deficit than conventional transcervical surgery. However, it is worth noting that postoperative hospitalization and the duration of drainage are much longer in robotic surgery than open surgery because of the extent of the flap. Moreover, the use of TORS for oropharyngeal minor salivary gland tumors, parotid gland tumors and sublingual gland ranulas was also reported by several surgeons, and the results showed favorable oncologic, surgical and functional outcomes, including no apparent neurovascular damage, a low positive margin rate and quick functional recovery, with excellent cosmetic results.^35, 36, 94, 95^

**Neck dissection.**

 Neck dissection followed by head and neck tumor removal is always necessary to reduce locoregional recurrence. Kang *et al.*^96^ first applied a robotic surgical system in a radical neck dissection *via* a transaxillary track for the staged treatment of thyroid carcinoma to avoid a long visible incision scar and muscle deformities in the neck area as well as to strengthen deep and corner dissections. However, the region of level I is hard to completely dissect *via* this approach. Therefore, to overcome the limitations mentioned above, robot-assisted radical or selective neck dissections *via* a retroauricular approach or a modified face-lift approach have been reported.^97, 98, 99, 100, 101, 102, 103, 104, 105, 106^ The results suggested that the robot-assisted surgery lasted longer than conventional surgery, but the intraoperative bleeding, lymph node retrieval, volume of drainage, hospitalization and related complications of robot-assisted neck dissection (RAND) were similar to those of open neck dissection. Furthermore, the patients who underwent robotic surgery were much more satisfied with the postsurgical aesthetics than those who underwent open surgery. Additionally, the study of Kim *et al.*^100^ and Tae *et al.*^105^ demonstrated that RAND may have a lower risk of lymphedema and lymph node recurrence than conventional neck dissection.

**Post-ablative defect reconstruction.**

 An extensive mucosal defect and, in some cases, direct orocervical fistula or pharyngocervical communication and exposure of the great vessels can result from en bloc resection of a head and neck neoplasm and subsequent or simultaneous neck dissection. Consequently, it is important to achieve a reliable reconstruction for these patients. The first use of a robotic surgical system in post-ablative defect reconstruction was reported by Genden *et al.*,^17^ in which a mucosal advancement flap, two pyriform mucosal flaps and three posterior pharyngeal wall flaps were performed. Since then, the robotic surgical system has been increasingly employed in head and neck defect reconstruction. Various flaps, including a mucosal muscle flap, radial forearm flap and free anterolateral femoral skin flap, were applied for reconstruction.^40, 60, 62, 107, 108^ All flaps survived, except for four mucosal muscle flaps in Genden *et al.*^107^ study. Moreover, the studies mentioned above also showed that robotic reconstruction surgery has a shorter operative time, better functional recovery and more satisfactory aesthetics than conventional surgery. Kim^109^ performed a mandibular reconstruction with a fibular flap using a robotic surgical system combined with simultaneous virtual surgical planning (VSP). His results indicated that robotic surgery with VSP may have a higher flap survival rate than conventional surgery, with less time and effort.

#### Cleft lip and palate

Currently, the use of robotic surgical systems in the treatment of cleft lip and palate is still in an early stage of development. Khan *et al.*^110^ first reported the theoretical feasibility of robotic intra-oral cleft surgery and Hynes pharyngoplasty in a pediatric airway manikin and human cadaver in 2015. In the same year, Nadjmi^111^ demonstrated the technical feasibility and safety of robot-assisted soft palate muscle reconstruction in 10 consecutive patients (mean age: 9.5 months) with palatal clefts after cadaveric TORS. The results showed that the surgical duration of TORS is much longer than conventional surgery; however, the hospital stays and functional recovery for the robotic approach were significantly shorter than for the manual approach. Nadjmi^111^ believed that this was because of the precise dissection provided by the robotic surgical system, which might reduce damage to the vascularization and related innervation of surrounding muscles.

#### Maxillofacial fracture

The management of bone fracture, similar to the robotic surgical system for fracture treatment, mainly consists of two procedures: reduction and fixation. However, the development of robotics for the treatment of fractures is much more difficult than in other regions for two main reasons. First, the position of fracture segments changes before and after reduction, making it difficult to provide precise navigation. Second, it is impossible to provide appropriate resistance during the fixation period because of the lack of tactile and haptic feedback. Therefore, improvements in the identification capability and mechanical properties of the surgical robot are anxiously awaited. Currently, several robotic surgical systems with an integrated force sensor were applied for arthroplasty, such as ROBODOC, Active Constraint Robot (ACROBOT) and Bone Resection Instrument Guidance by Intelligent Telemanipulator (BRIGIT). However, robotic fracture reduction and fixation are only used for long bone and pelvic fractures.^112, 113^ The clinical application of robotic surgical systems in maxillofacial fractures has not been reported.

#### Craniofacial asymmetry

The theoretical feasibility of robot-assisted orthognathic surgery was proposed in 2010 by Chen *et al.*,^114^ who suggested a method using the six degrees of freedom robot MOTOMAN to perform bone cutting and drilling based on the navigation system that they programmed. Later, Peking University developed a robotic surgical system for the design of orthognathic surgery, bone reconstruction and intraoperative navigation. However, the clinical application of robotic orthognathic surgery has not been reported, and the robotic surgical system mentioned above remains in an experimental stage.

#### OSAS

OSAS is the most common type of sleep apnea, resulting from complete or partial obstruction of the upper airway. It can be caused by decreased muscle tone, thickened soft tissue around the airway, such as nasal polyps or adenoid hypertrophy, and structural features, such as nasal septum deviation, which result in a narrowed airway. Continuous positive airway pressure (CPAP) was often used as a standard treatment for OSAS.^115^ For those OSAS sufferers unwilling or unable to comply with CPAP, a properly selected surgical treatment would be an alternative option, based on the patient’s-specific anatomy.^116^ Such treatments include tonsillectomy, uvulopalatopharyngoplasty (UPPP), reduction of the tongue base, maxillomandibular advancement and hyoid suspension. However, the BOT has important physiologic functions and has close contacts to surrounding muscles, vessels and nerves, and the conventional reduction of the BOT usually results in severe adverse postoperative reactions. Therefore, the robotic surgical system has emerged as a potential solution to this dilemma.

Vicini *et al.*^117^ reported the first application of TORS in the resection of the BOT, combined with conventional septoplasty, UPPP or supraglottoplasty, for OSAS patients in 2010 without any intraoperative and postoperative complications. The result showed a similar surgical duration to open surgery. No tracheotomy was required during surgery, and all patients had an excellent functional recovery. The postoperative Apnea–Hypopnea Index (AHI) and Epworth Sleepiness Scale (ESS) were significantly decreased from their preoperative values, and 90% of patients were satisfied with the results. Subsequently, TORS became widely applied for OSA sufferers for tonsillectomy, supraglottoplasty and glossectomy.^118, 119, 120, 121, 122, 123, 124, 125, 126, 127, 128^ Most of the studies demonstrated that TORS has a similar therapeutic efficacy and decreased postoperative pain, hospital stay and incidence of dysphagia compared with conventional surgery. Although almost all of the studies showed that the postoperative AHI, EES and snoring intensity are significantly improved by TORS, the cure rate still varies from 45 to 90%. Hoff *et al.*^122^ found that preoperative body mass index (BMI) may help the clinician predict the success of TORS; specifically, the cure rate is significantly higher in patients with BMI<30 than those with BMI>30. Moreover, when compared to submucosal minimally invasive lingual excision and radiofrequency BOT reduction, Friedman *et al.*^120, 121^ study indicated that robot-assisted partial glossectomy resulted in a greater AHI reduction, but longer functional recovery.

However, there are some specific adverse events that have been reported with TORS. A 12.5% transient dysgeusia rate was reported by Lee *et al.*^124^ in robotic lingual tonsillectomy; 3 of 12 patients complained of taste disturbance after robotic BOT resection in the study by Lin *et al.*,^125^ while 18.3% of patients experienced transient hypogeusia in Crawford *et al.*^126^ study after robot-assisted BOT resection. Toh *et al.*^127^ study showed that all patients experienced temporary anterior tongue numbness and temporary tongue soreness, while 35% of patients reported a temporary postoperative change in taste. Muderris *et al.*^128^ reported six cases of robotic lingual tonsillectomy, all of which had lingual edema. Lin and Crawford proposed that these complications might have resulted from the pressure of the tongue blade or mouth gag.

#### Others

**Laryngeal clefts and laryngocele**

Rahbar *et al.*^129^ described the application of TORS in five pediatric patients with laryngeal cleft after cadaver experiments. As a result, one patient with a type I laryngeal cleft and one with a type II cleft who underwent TORS for closure of the laryngeal cleft achieved great success without any intraoperative or postoperative complications. However, the surgical duration was much longer than conventional surgery because of the restriction of the surgical space; the surgical procedure failed to be completed in three patients because of limited transoral access. Ciabatti *et al.*^130^ used TORS for the excision of a large mixed laryngocele with short operative time and satisfactory aesthetics. No complications were observed, and an oral diet was started 1 day postoperatively and the patient was discharged 2 days after TORS.

**Ectopic lingual thyroid**

In May 2011, robot-assisted dissection of a lingual thyroid gland in three patients with minimal morbidity and excellent functional outcomes was successfully performed.^131^ Recently, an increasing number of ectopic lingual thyroids have been excised *via* a robotic surgical system.^43, 132, 133^ The results showed that patients undergoing TORS could start oral feeding on the first postoperative day, and no recurrence was observed within 2 months of follow-up. In Prisman *et al.*^133^ opinion, TORS should be regarded as a valid option for the treatment of ectopic lingual thyroid.

**Ptyalolithiasis**

Walvekar *et al.*^134^ first reported the successful removal of a 20-mm submandibular megalith and the subsequent repair of the salivary duct using a robotic surgical system. The total time involved was 120 min, and no complications were noted. Recently, Razavi *et al.*^135^ facilitated large submandibular gland stone removal using TORS in 22 patients. Procedural success was 100%, and no symptoms of recurrence or lingual nerve damage were recorded at follow-up. Meanwhile, they studied 135 patients who underwent TORS for removal of submandibular gland stones and showed that procedural success was reported in 75% of these patients; the lingual nerve damage rate was 2%.

**Vascular lesions**

Recently, the excision of BOT vascular lesions *via* a robotic surgical approach was described by Dziegielewski *et al.*,^136^ who found that it could be used in a safe manner to dissect BOT vascular lesions with maximum preservation of the surrounding vessels, nerves and muscles. Consequently, the postoperative damage to swallowing and speech function is minimal.

## Discussion

### Superiority and limitations

Robot-assisted surgery has been increasingly applied in the head and neck region and has ushered in a new era of MIS. Compared with conventional or endoscopic surgery, robotic surgery has several distinctive advantages and limitations (Table 1 and 2).

#### Superiority of robotic surgery

Magnified 3-dimensional visualization. The surgical space can be stereoscopic and 10–15 times magnified *via* two or more integrated cameras that are used in the system, which can enhance the surgeon’s capability to distinguish normal tissues from tumors and to preserve normal tissues to the highest extent. Thus, the tumor can be removed en bloc, with minimal morbidity and accelerated functional recovery.

Breaking the limit of human hands. The robotic arms are equipped with articulating surgical instruments, which provide increased degrees of freedom and extend the range of motion. As a result, the stability and accuracy of surgical procedures are improved.

Minimally invasive. A transcervical approach is often applied for the resection of head and neck neoplasms with or without mandibulotomy or a lip-splitting incision to obtain sufficient surgical space; this is accompanied by high morbidity and poor postoperative swallowing and speech functions. In contrast, robotic surgery could remove tumors *via* a minimally invasive approach, such as a transoral and a retroauricular approach, to decrease surgical complications and functional damage to a large extent. The average blood loss was minimal, and no patient required blood transfusions intra- or postoperatively.

Excellent manipulability. Remote operation and real-time shared surgery can be available *via* Internet and satellite technology.

Economizing medical staff. The robotic surgical system is highly automated; thus, only one surgeon, one anesthesiologist and one or two nurses are required, even for a difficult surgical operation. This could overcome the restrictions of operating room capacity and the shortage of medical resources.

#### Limitations of robotic surgery

Lack of tactile perception and proprioception. It is impossible, through a robotic surgical system, to feel the strength and resiliency of tissues or the radial pulse. Therefore, it is difficult to control bleeding in a timely fashion once exsanguinating hemorrhage occurs.

Lack of haptic feedback. For some fine motions, such as tying, suture breakage can occur as a result of excess tension. Additionally, several studies found that the postoperative rate of lingual edema is significantly higher with robotic surgery than with the conventional approach, as mentioned above, which may be due to long-term excess pressure. However, Hans *et al.*^32^ and several other researchers found that 3D visualization would compensate for the lack of haptic feedback, to some extent, with increased experience.

Complicated. The robotic surgical procedure is complicated and the operative duration is much longer than with open surgery. This is because the robot needs to be docked in an appropriate position before surgery, which requires additional time, especially in this early stage. With additional robotic surgery experience, the operative duration would be similar to open surgery.

Expensive. Cost is a major problem that limits its wide application. The primary expense of a single robotic surgical system, including installation, is ~1.5 million dollars, in addition to ~$100 000 for annual maintenance and ~$200 in disposable instruments per patient, which results in increased costs of surgery.^9^ In the short term, the robotic surgical system will not have a positive impact on cost because of several costs associated with systems, telecommunication, training personnel and infrastructure.^5^ However, several studies found that the reduction of related morbidity and hospitalization, and the decreased need for tracheotomy partially offset the additional cost engendered by robotic surgical systems.^33, 50, 52^

Large size. Robotic surgical systems are unwieldy and require considerable space. The bulky size of the instruments limits its application in the treatment of laryngeal carcinoma patients, who have limited mouth opening or mandibular retraction, and in transnasal surgeries or otology.

Lack of specific instruments for maxillofacial surgery. For instance, electric bone saws and drills. This problem will need to be resolved in the near future.

### Prospective of robotics in the head and neck region

The robotic surgical system is a novel, minimally invasive procedure with promising impact, and the development of robotic surgery is still in an early stage. There are several challenges and barriers to broader application and adoption of this technique. Further refinements are necessary before its wide application in maxillofacial surgery for head and neck neoplasms and in non-malignant diseases.

From a clinical perspective, the widespread use of robotic surgical systems in head and neck surgery is an inevitable development. The available research indicated excellent outcomes in terms of surgical morbidity, oncologic control and functional recovery for head and neck tumor patients treated by robotic surgical systems. However, there are several problems and uncertainties associated with robotic surgery. The incidence of capsule breakage or neoplasm fracture during robotic surgery is relatively high. Robotic surgery typically requires a long surgical duration or large storage of drainage, especially *via* a retroauricular approach or a modified face-lift approach, because of the extended flap. It remains unclear whether robotic surgery would improve the prognosis of HPV-negative patients. The regional or distant metastasis rate for robot-assisted resection of recurrent tumors is quite variable. However, because the robotic surgical system has been used for a relatively short time in the treatment of head and neck neoplasms, the problems mentioned above as well as the long-term effects and cost-effect analysis of this approach will require further study prior to it becoming a standard treatment paradigm. Particularly, specialization of robotic instruments for head and neck therapy, progressive miniaturization of its components, realization of haptic feedback, multisurgeon capability and flexible multiport access devices are anticipated for the future development of robotic surgery. Furthermore, VSP was reported to provide good guidance for robotic surgery, which will potentially enhance the accuracy and efficiency of robotic surgical systems. Therefore, a shorter surgical duration and superior reconstruction might be achieved when combining robotic surgery and VSP; this approach is another anticipated trend in robotic surgery in the future (Table 1).

Regarding other applications in the head and neck, robotic surgery has been widely used in OSAS patients, and it is undoubtedly a promising approach for those who cannot tolerate CPAP. However, the success rate remains unsatisfactory, possibly because of the nature of the multiple risk factors for OSAS. Therefore, robotic surgery for OSAS should only be used after careful patient selection regarding severity, age, BMI and related soft tissue structures. Furthermore, the combination of robotic resection of the BOT and conventional UPPP or sphincter pharyngoplasty might be a rational operation in the future. Moreover, it is almost impossible to use a robotic surgical system in the treatment of maxillofacial fractures and craniofacial asymmetry owing to the current lack of tactile and haptic feedback. Specifically, an appropriate resistance is not provided by current robotic surgical technology to prevent additional damage when performing a fracture reduction or an osteotomy. More work needs to be done, from theoretical feasibility to the clinical application of robotic surgical systems, in the management of maxillofacial fractures and craniofacial asymmetry. Additionally, the available studies that used robotic surgery in the treatment of lip and palate patients are quite limited. Although the only clinical research demonstrated significantly shorter hospital stays and better functional recovery than conventional surgery because of the precise dissection and reconstruction in robotic surgery, further studies with larger samples are still of paramount importance to ensure the safety and feasibility of robot-assisted surgery for cleft lip and palate patients. Similarly, the long-term effectiveness and safety of robotic surgery applied in other conditions, such as ectopic lingual thyroid and ptyalolithiasis, also require further study. Furthermore, selection of surgical procedures appropriate for the system is a challenge as well, except for the requirement of more well-designed studies. Standard surgical procedures permit the application of surgical robots. The diversity of maxillofacial surgery (that is, cleft lip surgery) set the development of robotic surgery back to a certain extent, and these procedures should be standardized before surgical robots are widely applied. In addition, although oscillating and surgical drills were applied in robotic arthroplasty, a similar application suitable for maxillofacial surgery has not been pursued. To summarize, instrument specialization, the realization of more precise intraoperative navigation, and further applications with large samples in various maxillofacial surgeries will all further the development of robotic surgery in the treatment of non-malignant craniofacial conditions (Table 2).

From a technical perspective, the considerable operative duration is currently one of the main deficiencies of robotic surgery because of extended times for robot docking, changing tools and inserting supplies. To address this deficiency, two technical projects were recently proposed.^5^ One is “Robotic systems,” which integrates multiple surgical robots into a single “robotic cell.” A robotic tool changer or a robotic supply dispenser may perform the function instead of nurses when a different tool is needed during an operation in the future. The other is “automatic or autonomous surgery.” To perform a pre-programmed task under an unstructured environment in a living system is difficult because of the greater variability, but it is theoretically realizable by collecting large amounts of previously “rehearsed” and “saved” surgical procedures. In addition, a lack of tactile and haptic feedback is an important deficiency of a robotic surgical system as well. Haptic feedback provides an operator with both sense and interaction with an interface. Haptic feedback can help prevent inadvertent damage to normal tissues and distinguish specific tissues features, such as cardiac arteries. Today’s operating instruments in robotic systems are all simple mechanical devices; the surgeon could only proceed to dissect depending on the subjective sense of touch *via* visualization. There is no suitable haptic sensor that is incorporated with current robotic surgical system, although several related mechanical sensors have been investigated. Tsang^137^ determined that VerroTouch, an early add-on, including a sensor placed on the robotic instrument and a vibration actuator fixed on the handle to provide haptic feedback, is capable of solving this problem, but none found it essential. In orthopedic surgery, several robotic systems, such as ACROBOT and MAKO RIO, were reported to have the ability to realize haptic feedback during the execution phase of arthroplasty by constraining the surgeon to operate within a predefined safe region. Once the surgeon attempts to operate outside the boundary, the control systems and drive systems inside the manipulator apply resistance to the motion to keep the effector within the predefined surgical plan.^5^ With the development of Computer-Aided Manufacturing/Computer-Aided Design in maxillofacial surgery, a similar technique to MAKO RIO could be applied for head and neck disease soon. Additionally, there are a number of other engineering barriers have to overcome, including: (1) ease of use: the current robotic surgical systems always have a high level of complexity and require advanced training, which may cause some highly specialized surgeons to shy away from these procedures; (2) reliability of telecommunication: low packet loss and limited latency are of great importance for consistently and safely operating at a distance.

## Conclusion

The primary outcomes of robotic surgery in the head and neck region demonstrate good disease control, quick postoperative functional recovery and low surgical morbidity. However, definitive recommendations for the application of robotic surgical systems in the treatment of head and neck tumors, cleft lip and palate, OSAS and other conditions will require more well-designed studies and technical modifications in current surgical robots and in the future.

## Figures and Tables

**Table 1:** Current application and future development of robotic surgery in head and neck neoplasms

Patients	Superiority	Limitations	Future development
Head and neck neoplasms resection			
Upper aerodigestive tract tumor^16–65^	In common: decreased damage to surrounding tissues; superior function recovery, better oncologic control and lower morbidity than conventional open surgery as well as radiochemical therapy; excellent aesthetics	In common: long surgical duration; lack of specific instruments (sharp instrumentation); lack of haptic feedback, and expensive	In common: realization of haptic feedback; bimanual operation and improvement of sharp instruments
Parapharyngeal spcae tumor^36, 61, 73–75^ Thyroid gland tumor and mediastinal parathyroid^77–89^	Upper aerodigestive tract tumor: high effectiveness in detection of unknown primary tumors	Thyroidectomy: long hospitalization and considerable duration of drainage	Thyroidectomy: modified surgical approach to reduce the extent of the flap
Salivary glands tumor^90–95^			
Neck dissection^96–106^	Thyroidectomy: easy to ligate the tract after carefully tracing it		Flap reconstruction: combination of robotic surgery and virtual surgical planning
Post-ablative defect reconstruction^17, 40, 60, 62, 107–109^	Neck dissection: low risk of lymph-edema and lymph node recurrence		
	Flap reconstruction: high survive rate		

**Table 2:** Current application and future development of robotic surgery in head and neck non-malignant diseases

Patients	Superiority	Limitations	Future development
Lip and palate cleft^110–111^	Low damage to the vascularization and related innervation of surrounding muscles, quick function recovery	Long surgical duration	More high-quality clinical investigation
Maxillofacail fracture	Insufficient data	Insufficient data	Specific design of related robotic surgical system
Craniofacial asymmetry^114–115^	Insufficient data	Insufficient data	Transition from theoretical feasibility to clinical application
OSAS^117–128^	Low intropetative bleeding and tracheotomy, decreased postoperative pain, hospital stay as well as incidence of dysphagia	Unstable cure rate varies from 45% to 90%, significant postoperative lingual oedema and transient hypogeusia	Combination of robotic resection of BOT and conventional surgery like uvulopalatopharyngoplasty or sphincter pharyngoplasty
Others			
Laryngeal clefts^129^	In common; minimal damage to surrounding normal tissues as well as speech and swallow function; excellent aesthetics	Laryngeal lefts: unsatisfactory cure rate	Laryngeal lefts: application of specific miniaturized instruments to obtain enough surgical space
Laryngocele^130^	Laryngocele: short operative time		
Ectopic lingual thyroid^131–133^	Ectopic lingual thyroid: short operative time and low recurrence		
Ptyalolithiasis^134–135^	Ptyalolithiasis: high cure rate and low lingual nerve damage rate		
Vascular lesion^136^			

OSAS, obstructive sleep apnea syndrome.
